# Effects of Delayed Harvest and Additives on Fermentation Quality and Bacterial Community of Corn Stalk Silage

**DOI:** 10.3389/fmicb.2021.687481

**Published:** 2021-07-07

**Authors:** Linna Guo, Yongxiang Lu, Ping Li, Liangyin Chen, Wenlong Gou, Changbin Zhang

**Affiliations:** ^1^College of Animal Science, Guizhou University, Guiyang, China; ^2^College of Grassland Science and Technology, China Agricultural University, Beijing, China; ^3^Sichuan Academy of Grassland Sciences, Chengdu, China

**Keywords:** corn stalk silage, delayed harvest, sodium benzoate, *Lactobacillus plantarum*, bacterial community

## Abstract

This study aimed to investigate the effects of delayed harvest and additives on the fermentation quality and bacterial community of corn stalk silage in South China. The corn stalks after ear harvest at the 0 day (D0), 7 days (D7), and 15 days (D15) were used to produce small-bale silages. The silages at each harvest time were treated without (control, CK) or with *Lactobacillus plantarum* (LP) and sodium benzoate (BF). The results showed that delayed harvest increased pH and acetic acid content and reduced lactic acid content in corn stalk silage (*p* < 0.05). Compared with CK, the additives decreased the contents of butyric acid and ammonia nitrogen (NH_3_-N; *p* < 0.05). The silage treated with LP increased the content of lactic acid and decreased pH (*p* < 0.05); the silage treated with BF decreased counts of coliform bacteria and yeasts and increased residual water soluble carbohydrates (WSC) content (*p* < 0.05). Single Molecule, Real-Time sequencing (SMRT) revealed that the abundance of *L. plantarum* increased, while the abundance of *Lactobacillus brevis* and *Lactobacillus ginsenosidimutans* decreased with the delayed harvest. Additives influenced the bacterial community structure of corn stalk silage, revealed by enhanced bacterial diversity on D0 and reduced on D7 (*p* < 0.05). Our research indicated that delayed harvest could exert a positive effect on acetic acid production, and additives could inhibit the butyric acid fermentation and protein degradation of corn stalk silage by shifting bacterial community composition.

## Introduction

Corn (*Zea mays*) is an important grain-forage crop. In recent years, the intercropping technology has been widely applied to produce abundant corn stalk in South China. However, most corn stalks with a high content of structural carbohydrates (50–70%) are discarded, which is wasteful and creates environmental atmospheric pollution (Li et al., 2015; Zhang et al., 2020a). Furthermore, with the increase of the demand for animal products, feed shortage hinders the development of the animal husbandry. Nowadays, high stay-green corn has been developed, which is characterized by a high chlorophyll concentration in the leaves at “stalk” maturity, with high stalk and leaf moisture concentrations (Thomas and Smart, 1993; Bekavac et al., 1998). Importantly, moisture and crude protein (CP) concentration are relatively high in stay-green corn stalks. It is cost-effective to convert stay-green corn stalk into ruminal feed. However, how to effectively utilize corn stalks in the animal production system is a concerned issue.

Ensiling has been regarded as an effective method for preserving fresh forage due to its long storage duration, good palatability, and high nutrition. Traditional corn is usually harvested at milk ripe or wax stages for producing high-quality silage (Zhang et al., 2010; Wang et al., 2018a). In practice, delayed harvest reduced feed value of corn stalk (Cai et al., 2020). To optimize the storage of corn stalks, *Lactobacillus plantarum* (LP), a homo-fermentative strain of lactic acid bacteria (LAB), has been used as additive to enhance the fermentation quality of silage (Ni et al., 2017a; Xu et al., 2018; Guo et al., 2020a,b). Recently, sodium benzoate (BF) has been mainly applied as a microbial growth controller in silage production (Zhang et al., 2020b), which could inhibit the growth of harmful bacteria and fungi, thus improve the fermentation quality and aerobic stability of silages (Kleinschmit et al., 2005; Da Silva et al., 2015; Muck et al., 2018). However, there are only a few literatures on the efficiency of both additives (LP and BF) in producing silage of stay-green corn stalks, especially during delayed harvest in the southeast of Qinghai Tibetan Plateau.

In recent years, next-generation sequencing (NGS) technology has made the microbiota classification accurate to genus and has been increasingly utilized to analyze microbial population in silage (Ni et al., 2017a; Wang et al., 2018b; Keshri et al., 2019). However, it restricts the sensitivity and accuracy of classification. Nowadays, Single Molecule, Real-Time sequencing (SMRT) is used for high taxonomic resolution at the species level and has been employed in silages of corn, Italian ryegrass, and paper mulberry (Xu et al., 2018; Yan et al., 2019; Du et al., 2021). Nevertheless, there are few reports investigating the bacterial community based on species level and the mechanism underlying the response to additives of delayed harvest corn stalk silage.

In this study, we supposed that delayed harvest could influence the ensilability of corn stalk, and the use of additives during ensiling were beneficial to fermentation quality of the silage mainly by shifting bacterial community compositions. Therefore, we aimed to investigate the effects of delayed harvest and additives on the bacterial community and fermentation quality of corn stalk silage.

## Materials and Methods

### Silage Preparation

Eighteen field plots were designed to cultivate stay-green corns (Tieyan 53, Beijing Hejiayun Agriculture Sci-Tech Co., Ltd.) on the experimental base of Sichuan Academy of Grassland Sciences, Aba, China (N 31°51'-33°33', E 101°51'-103°22'). Stay-green corn stalks were cut after 0, 7, or 15 days of ear harvest (six plots for each delayed harvest; ear provided for human diet; and assessed at D0, D7, and D15, respectively), and chopped to a length of 0.5–1.0 cm. In each plot, the chopped stalks were randomly divided into three equal parts for treatment with additive. The ensiling materials were treated with no additive as control (CK), with LP [a recommended application rate of 10^5^ cfu/g fresh matter (FM), isolated from corn silage in our laboratory and was reported from Chen et al., 2020a,b] or with BF (a suggested application rate of 1.0 g/kg FM, provided from Prodpad Technology Co., Ltd.). Each bale (about one cubic meter) contained approximately 800 kg (FM) of corn stalks, and a total of 54 bales (3 delayed harvest × 3 treatments × 6 replicates) were produced using small baling system, with the density of about 580 kg/m^2^ and the blank stretch film of four layers. All bales were stored at room temperature (10–20°C) for 60 days, and then sampled for the analysis on chemical, microbial composition, fermentation parameter, and bacterial community.

### Chemical Analysis

Each sample of 20 g was mixed with 180 ml sterile water and a laboratory juicer for 1 min, and then filtered through four layers of cheesecloth. The filtrate was subjected to centrifugation (4,500 × *g*, 15 min, 4°C). The supernatant was used to measure pH, NH_3_-N, and organic acid. The pH was determined by pH meter. NH_3_-N was determined by method of Broderick and Kang (1980). Lactic, acetic, propionic, and butyric acids were analyzed using high-performance liquid chromatography (Tian et al., 2017).

Each sample of 200 g was dried at 65°C for a constant weight to determine dry matter (DM) content, and then ground by a 0.20 mm sieve for the following analysis. CP was determined by the method of AOAC (1990). Both neutral detergent fiber (aNDF, neutral detergent fiber assayed with a heat stable amylase and expressed inclusive of residual ash) and acid detergent fiber (ADF) were determined using an Ankom 2000 fiber analyzer (Ankom Technology, Fairport, NY) by method of Van Soest et al. (1991). WSC was determined by the method of Murphy (1958).

### Microbial Analysis

The microbial count of each sample was determined by the method of Cai et al. (1999), and described by Li et al. (2020). In brief, each sample of 10 g was mixed with 90 ml sterile saline, shaken for 30 min and then filtered through sterile gauze. Serial dilutions were performed. The count of LAB was determined on MRS agar (CM188, Land Bridge Technology Co., Ltd., Beijing, China) and incubated at 30°C for 48–72 h under anaerobic conditions (Anaerobic box; TEHER Hard Anaerobox, ANX-1; Hirosawa Ltd., Tokyo, Japan). Aerobic bacteria were counted on nutrient agar (CM107, Land Bridge Technology Co., Ltd., Beijing, China) and inoculated at 28°C for 24–36 h under aerobic conditions. Coliform bacteria were counted on ECCA cheomogenic medium (RP0436, Guangzhou LES Biological Technology, Co. Ltd., Guangzhou China). Yeasts and molds were counted on malt extract agar with 1.5 mg/L Tetracycline (CM164, Land Bridge Technology Co., Ltd., Beijing, China) and incubated at 28°C for 48 h. Yeasts were distinguished from molds through colony appearance and observation of cell morphology.

Total genome DNA from each sample was extracted by CTAB method. DNA after purification was diluted to 1 ng/ml using sterile water. The full-length 16S ribosomal RNA (rRNA) gene was amplified used specific primer (27F and 1541R) with the barcode (Yan et al., 2019). The PCR reaction was carried out by TransStart®FastPfu DNA Polymerase (TransGen Biotech). Triplicate amplifications from each sample were mixed for establish libraries. PCR products were mixed in equal density ratios and purified with QIAquick@Gel Extraction Kit (QIAGEN). Libraries were established using SMRTbellTMTemplate Prep Kit (PacBio) following manufacturer’s recommendations, and then sequenced on the PacBio Sequel platform.

Raw sequences were initially processed through the PacBio SMRTportal. Sequences were filtered to produce reads without barcode and primer sequence. The reads were compared with the reference database using UCHIME algorithm^1^ to detect chimera sequences (Edgar et al., 2011), and then the chimera sequences were removed for obstaining clean reads (Haas et al., 2011). Sequences analysis was performed by Uparse software (Uparse v7.0.1001; Edgar, 2013).^2^ Sequences with the similarity ≥97% were distributed to the same operational taxonomic unit (OUT).

Following the OTU analysis, principal coordinates analysis (PCoA) was performed using R software (Version 2.15.3) based on the beta-diversity analysis. Representative sequence for each OTU was screened out for annotating taxonomic information in the SSUrRNA Database of Silva Database (Qiong et al., 2007; Christian et al., 2012). After the establishment of the phylogenetic relationship (Edgar, 2004), the number of observed species, richness index of abundance-based coverage estimator (ACE) and Chao 1, and diversity index of Shannon were calculated using QIIME software (Version1.9.1) and displayed with R software (Version 2.15.3). The heat map of spearman analysis was performed using a R based statistics tool.

### Statistical Analysis

Before statistical analysis, microbial counts of each silage sample were estimated as log_10_ cfu/g of FM. Factorial analysis of variance was performed to evaluate the effects of delayed harvest (D), additive (A), and their interaction (D × A) on the chemical composition, microbial population, and bacterial community indices of silage in the General Line Model of SPSS (SPSS 25.0 program, SPSS Inc., Chicago, Illinois, United States). There were significant differences only when the probability level was lower than 0.05 (*p* < 0.05).

## Results and Discussion

### Chemical and Microbial Compositions of Corn Stalks Prior to Ensiling

Chemical and microbial compositions of stay-green corns prior to ensiling were shown in Table 1. Delayed harvest had significant effects on contents of DM, WSC, CP, aNDF, ADF, and counts of LAB, coliform bacteria, aerobic bacteria, and yeasts (*p* < 0.05). DM, aNDF, and ADF contents of corn stalks significantly increased with the harvest delayed (*p* < 0.05), which indicated that the lignification degree of corns could be deepened with delayed harvest. WSC and CP contents in this study significantly decreased from 8.88 to 7.29% DM and from 9.22 to 6.53% DM, respectively. There was also similar observation from Guo et al. (2019), which suggested a loss of nutrient in delayed harvest corn stalk. WSC is an important substance for the growth and propagation of epiphytic microorganisms, especially for LAB. In this study, the WSC content beyond 6% DM was sufficient for the production of high-quality silage. However, relatively high counts (10^5^–10^7^ cfu/g FM) of undesirable microorganisms (coliform bacteria, aerobic bacteria, and yeasts) on the plants were observed on corn stalks from D0 to D15. Although the count of epiphytic LAB on the plant of direct-cut stay-green corn (D0) was 10^5^ cfu/g FM, which was enough for initiating lactic acid fermentation under anaerobic condition. The count decreased from D0 to D15 to 10^4^ cfu/g FM, which was insufficient for silage preservation (Cai et al., 1999). The above findings indicated that the delayed harvest could be harmful for the fermentation quality of corn stalk silage, and the unfavorable properties of stay-green corns for silage implied that it was necessary to add exogenous additives to enhance fermentation during ensiling.

**Table 1:** Chemical and microbial compositions of corn stalks after 0 (D0), 7 (D7), and 15 (D15) days of ear harvest.

Delayed harvest	DM	WSC	CP	aNDF	ADF	Lactic acid bacteria	Coliform bacteria	Aerobic bacteria	Yeasts	Molds
	%	% DM				Log_10_ cfu/g FM				
D0	25.31^c^	8.88^a^	9.22^a^	50.41^b^	31.18^^b^^	5.35^a^	6.50^a^	7.48^a^	6.11^b^	3.52
D7	28.46^b^	8.15^a^^b^	7.01^b^	58.79^a^	32.19^b^	4.87^b^	5.23^b^	7.16^a^	6.68^a^	3.44
D15	35.18^a^	7.29^b^	6.53^c^	61.48^a^	34.01^a^	4.19^c^	5.76^b^	5.45^b^	6.82^a^	3.69
SEM	1.49	0.35	0.42	1.69	0.47	0.18	0.20	0.33	0.12	0.09
Significance (*p*-value)	<0.001	0.044	<0.001	<0.001	0.007	0.001	0.003	<0.001	0.006	0.658

DM, dry matter; WSC, water soluble carbohydrates; CP, crude protein; ADF, acid detergent fiber; aNDF, neutral detergent fiber; FM, fresh matter; and SEM, standard of error mean. Different letters within a column are significantly different (*p* < 0.05).

### Chemical Composition of Corn Stalk Silage

The chemical composition of corn stalk silages treated without (CK) or with LP and BF was shown in Table 2. Delayed harvest significantly affected all parameters of chemical composition (*p* < 0.001). Additives had significant effects on residual WSC, aNDF, and ADF contents (*p* < 0.05). Their interaction only had a significant effect on residual WSC and aNDF contents of silage (*p* < 0.05). DM, aNDF, and ADF contents of silage significantly increased with advancing maturity, which might due to the decrease of the moisture and the increase of the cell wall content in stem with advancing maturity (Yari et al., 2012; Sikora et al., 2019). The CP content of corn stalk silage gradually decreased (*p* < 0.001) from D0 to D15, which might due to the higher protein content in corn leaves than stems. Moreover, a high CP content was found in LP-treated silage numerically. The behind reason might be that proteolysis was inhibited by acid accumulation during ensiling (Chen et al., 2020b). During ensiling, some microbes, such as coliform bacteria, LAB, and yeasts, could produce energy with WSC (Kung et al., 2018; Queiroz et al., 2018). In this study, a high residual WSC content (3.48–4.64% DM) was observed in BF-treated silages on D0 and D15 (*p* < 0.05). Similar reports were also found from Alli et al. (1985) and Da Silva et al. (2015), and that might because the antimicrobial capacity of BF could inhibit the growth of microbe, and then reduce their WSC metabolism. This confirmed that the treatment with LP and BF could enhance the preservation of silage nutrients although there was a loss of nutrients with the delayed harvest.

**Table 2:** Chemical composition of corn stalk silages treated without (CK) or with *Lactobacillus plantarum* (LP) and sodium benzoate (BF).

Delayed harvest	Additive	DM	Residual WSC	CP	aNDF	ADF
		%		% DM		
D0	CK	23.89	4.02^bc^	8.59	52.75^f^	34.17
	LP	22.20	4.32^ab^	9.02	56.66^e^	33.11
	BF	22.93	4.64^a^	8.58	53.94^f^	32.58
D7	CK	26.01	2.78^e^	6.09	62.15^bc^	34.12
	LP	27.81	3.67^cd^	6.11	61.16^cd^	33.28
	BF	26.76	3.48^d^	6.10	59.01^d^	33.03
D15	CK	32.04	2.35^f^	5.24	64.84^a^	36.32
	LP	34.05	2.30^f^	5.44	65.44^a^	36.92
	BF	32.46	3.94^bc^	5.42	63.77^ab^	35.88
SEM		4.37	0.12	0.21	0.65	0.24
Significance (*p*-value)						
Delayed harvest		<0.001	<0.001	<0.001	<0.001	<0.001
Additive		0.581	<0.001	0.402	0.008	0.014
Delayed harvest × additive		0.762	<0.001	0.760	0.034	0.313

DM, dry matter; Residual WSC, residual water soluble carbohydrates; CP, crude protein; ADF, acid detergent fiber; aNDF, neutral detergent fiber; and SEM, standard of error mean. D0-15 indicated stay-green corn stalks after 0, 7, and 15 days of ear harvest. Different letters within a column are significantly different (*p* < 0.05).

As shown in Table 3, the interaction of delayed harvest and additives significantly affected final pH and the contents of NH_3_-N, lactic acid, acetic acid, and butyric acid (*p* < 0.05), in which delayed harvest had significant effects on all the fermentation indicators in Table 3 (*p* < 0.05), and additives significantly affected final pH and the contents of NH_3_-N, lactic acid, and acetic acid (*p* < 0.001). According to Kung et al. (2018), the concentrations of lactic acid and acetic acid were usually negatively correlated to DM content. Our study showed that delayed harvest significantly decreased the lactic acid content (*p* < 0.001), but increased acetic acid content with the increase of DM content (*p* < 0.001). The probable cause was that delayed harvest promoted the metabolism of some acetic acid-producing microbes.

**Table 3:** Final pH, NH_3_-N, and fermentation acids of corn stalk silages treated without (CK) or with LP and BF.

Delayed harvest	Additive	Final pH	NH_3_-N	Lactic acid	Acetic acid	Propionic acid	Butyric acid
			% total N	% DM			
D0	CK	3.73^e^	13.32^a^	2.60^ab^	0.15^e^	0.12	0.19^a^
	LP	3.70^e^	8.77^c^	2.79^a^	0.19^de^	0.14	0.11^b^
	BF	3.89^d^	8.91^c^	2.70^a^	0.17^de^	0.14	0.13^b^
D7	CK	4.41^a^	13.41^a^	1.04^e^	0.38^b^	0.05	0.13^b^
	LP	4.11^c^	10.21^b^	2.40^b^	0.23^c^	0.07	0.01^c^
	BF	4.31^ab^	10.21^b^	0.96^e^	0.22^cd^	0.06	0.01^c^
D15	CK	4.20^bc^	10.80^b^	1.30^d^	0.46^a^	0.14	0.01^c^
	LP	4.18^bc^	10.57^b^	2.06^c^	0.37^b^	0.15	0.01^c^
	BF	4.36^a^	8.94^c^	1.33^d^	0.40^ab^	0.10	0.01^c^
SEM		0.27	0.25	0.06	0.02	0.04	—
Significance (*p*-value)							
Delayed harvest		<0.001	<0.001	<0.001	<0.001	<0.001	0.007
Additive		<0.001	<0.001	<0.001	<0.001	0.393	0.428
Delayed harvest × additive		0.028	<0.001	<0.001	<0.001	0.322	0.006

DM, dry matter; NH_3_-N, ammonia nitrogen; and SEM, standard of error mean. D0-15 indicated corn stalks after 0, 7, and 15 days of ear harvest. Different letters within a column are significantly different (*p* < 0.05).

When the DM content of fresh forages was above 30–35% DM and induced the rapid production of lactic acid, clostridial fermentation could be minimized because clostridia were intolerant of high osmotic pressure and low pH, thus resulting in a content of low butyric acid (Kung et al., 2018). In our study, the content of butyric acid decreased with the harvest delayed (*p* < 0.05), due to the increase of DM content. Additives significantly reduced the content of butyric acid on D0 and D7 (*p* < 0.05). This might be due to the lower pH in LP-treated silage and the antibacterial ability of BF. NH_3_-N is an important indicator for CP degradation, and its content in well-preserved silage should be lower than 10% total N (Chen et al., 2020b; Cheng et al., 2021). However, the NH_3_-N content of CK on each harvest day was higher than that level. The addition of BF and LP reduced the NH_3_-N content in silages on D0 and D7 compare with the CK samples (*p* < 0.05). Furthermore, the NH_3_-N content of LP-treated silage on D0 and BF-treated silages on D0 and D15 were below 10% total N, which suggested their low protein proteolysis.

Delayed harvest restricted the reduction of pH value, which was similar to Wang et al. (2018a), because high DM and low moisture contents in corn stalks after delayed harvest inhibited the silage fermentation. In general, the pH required for successful ensiling was lower than 4.2. In this study, the pH in the LP-treated silages was below 4.2, which might indicate that the addition with LP could help inhibit the unfavorable factors produced by the lower fermentation quality due to delayed harvest. Compared with CK samples, the treatment with LP significantly increased the content of lactic acid in silages on D7 and D15 (*p* < 0.05). Therefore, LAB inoculation might help promote rapid and vigorous fermentation of corn stalks. Studies from Kleinschmit et al. (2005), Teller et al. (2012), and Da Silva et al. (2015) showed that the addition of BF during ensiling had no effect on the concentrations of lactic acid in corn silages. In this study, the BF-treated silage had the highest pH value and the lowest content of lactic acid (*p* < 0.05), which further confirmed that BF might inhibit the activity or growth of microbial groups, thus reducing the fermentation to acid production. This indicated that good nutrient preservation of LP‐ or BF-treated silages were attributed to different fermentation qualities. Inoculation of LP during ensiling exerted a good performance in improving silage fermentation, whereas the use of BF during ensiling reduced NH_3_-N by limiting the microbial fermentation.

### Microbial Population of Corn Stalk Silage

**Figure 1:**
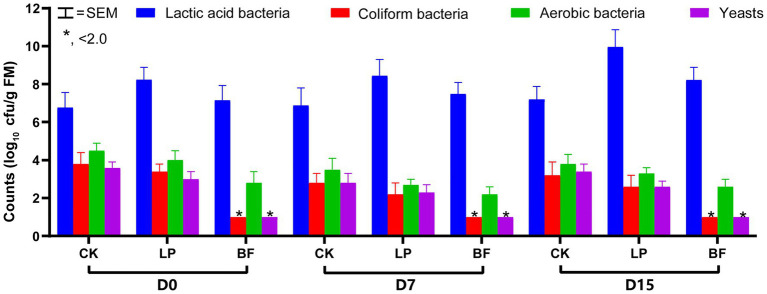
Microbial composition of corn stalk silages treated without (CK) or with *L. plantarum* and BF. D0, D7, and D15 indicated corn stalks after 0, 7, and 15 days of ear harvest; SEM, standard of error mean; ^*^significance at *p* < 0.05.

The silage fermentation process is initiated and controlled with microorganisms. As shown in Figure 1, all silages showed a similar microbial composition by plate culture. The counts of yeasts and coliform bacteria only in BF-treated silages were below the detected level (<2.0 log_10_ cfu/g FM). The inhibition of yeasts in BF-treated silages might involve some mechanisms of defect in amino acid uptake, such as the failure of enzymatic systems in the glycolysis and citric acid cycle in yeast cell (Santos et al., 2019). The similar results occurred in grain and corn silages (Knicky and Spörndly, 2011; Morais et al., 2017; Santos et al., 2019). According to Kung et al. (2018), coliform bacteria and yeasts could compete with LAB for fermentation substrates. Our findings indicated that the treatment with BF was useful to inhibit yeasts and coliform bacteria to create the condition where LAB population could grow and propagate quickly.

### Bacterial Community Indices of Corn Stalk Silages

The third generation Pacific Biosciences (PacBio) SMRT improves the classification sensitivity and accuracy of microbial community in silages (Xu et al., 2018). In order to further investigate the bacterial community of corn stalk silage after delayed harvest in South China, SMRT sequencing was used to describe them.

The bacterial alpha diversity of silage samples was shown in Table 4. The PE reads of each sample ranged from 5,847 to 6,693, and the total number of observed species was 875. The delayed harvest, additives and their interactions significantly affected the bacterial richness (Chao 1 and ACE) and the diversity (Shannon and PD whole tree; *p* < 0.05). Compared with CK samples, additives increased Chao 1, ACE, Shannon, and PD whole tree of silages on D0 (*p* < 0.05). However, the reverse trend was observed in silages at D7 and D15. This might be due to the fact that the use of additives enhanced the superiority of dominant bacteria and subsequently reduced the richness and diversity of other bacteria in silages with a high DM (Ni et al., 2017a). Especially, the treatment with LP had the lowest Shannon and PD whole tree in silages on D7 and D15 (*p* < 0.05). According to Kung et al. (2003) and Oliveira et al. (2017), the inoculation with exogenous LAB generally had a positive effect on sufficient lactic acid production and inhibited the activity of other harmful bacteria during the fermentation process. The findings of our study might confirm that exogenous *L. plantarum* exhibited high competitiveness for dominance during silage fermentation of corn stalks. Therefore, it is necessary to investigate the dominated species of bacterial community of corn stalks.

**Table 4:** Bacterial alpha-diversity of corn stalk silages treated without (CK) or with *L. plantarum* and BF.

Delayed harvest	Additive	PE reads	Observed species	Shannon	Chao 1	ACE	PD whole tree
D0	CK	6,050	110^bc^	2.27^b^	214.00^d^	239.01^d^	8.67^cd^
	LP	6,043	221^a^	2.76^a^	492.73^b^	594.48^b^	12.94^a^
	BF	6,185	157^ab^	2.76^a^	323.83^c^	397.76^c^	10.58^b^
D7	CK	6,428	134^b^	1.76^c^	576.85^a^	747.02^a^	9.73^bc^
	LP	5,847	37^c^	0.91^e^	51.87^f^	55.06^f^	4.95^fg^
	BF	6,165	86^bc^	1.21^d^	130.29^e^	150.80^e^	8.27^d^
D15	CK	6,390	55^c^	2.26^b^	64.30^f^	68.71^f^	6.36^e^
	LP	6,693	30^c^	1.16^d^	37.76^f^	38.23^f^	4.39^g^
	BF	6,049	45^c^	1.88^c^	54.22^f^	58.76^f^	6.01^ef^
SEM		257	9	0.09	26.58	34.06	0.39
Significance (*p*-value)							
Delayed harvest		0.327	<0.001	<0.001	<0.001	<0.001	<0.001
Additive		0.720	0.704	<0.001	<0.001	<0.001	0.031
Delayed harvest × additive		0.443	<0.001	<0.001	<0.001	<0.001	<0.001

SEM, standard of error mean. D0-15 indicated stay-green corn stalks after 0, 7, and 15 days of ear harvest. Different letters within a column are significantly different (*p* < 0.05).

**Figure 2:**
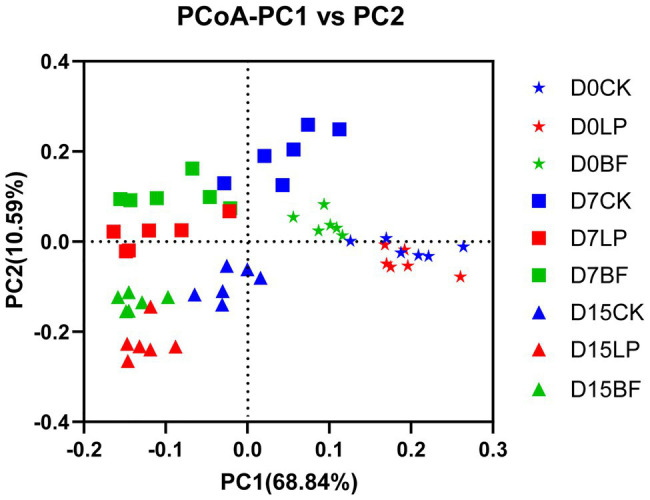
Principal coordinates analysis (PCoA) analysis of bacterial community structure of corn stalk silages treated without (CK) or with *L. plantarum* and BF. D0, D7, and D15 indicated stay-green corn stalks after 0, 7, and 15 days of ear harvest.

As shown in Figure 2, PCoA revealed that component 1 and component 2 could explain 68.84 and 10.59% of the total variance in bacterial community structure, respectively. Silage samples were well separated between D0, D7, and D15, which suggested that bacterial community would be affected by harvest time of corn stalks. Report from Ni et al. (2017b) showed that the variation of microbial community might explain the differences of silage quality. The findings of our study revealed that additives increased the dissimilarity in bacterial community in silage samples on D7 and D15, suggesting that the treatment with LP and BF could markedly shift the bacterial community composition and construction.

**Figure 3:**
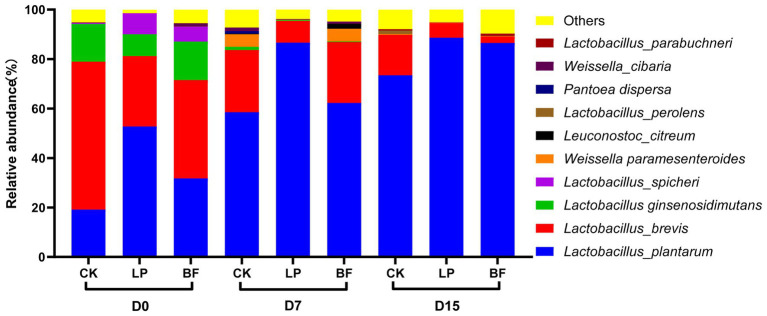
Relative abundance of top 10 bacterial species in corn stalk silages treated without (CK) or with *L. plantarum* and BF. D0, D7, and D15 indicated corn stalks after 0, 7, and 15 days of ear harvest.

The relative abundance of the top 10 bacterial species in corn stalk silage was shown in Figure 3. *Lactobacillus plantarum* and *Lactobacillus brevis* were the dominant species in corn stalk silages. With the delayed harvest, the abundance of *L. plantarum* increased, while the abundance of *L. brevis* decreased. It is generally recognized that *L. plantarum* is often used as an inoculant to promote silage fermentation (Yan et al., 2019). However, the increased dominance of *L. plantarum* was not effective in enhancing the content of lactic acid with the delayed harvest, because the low moisture content inhibited the activity of most microorganisms, and dominant *L. plantarum* showed a high survival competitiveness but a limited fermentation capacity on inferior condition. Compared with CK samples, the abundance of *L. brevis* was lower, while the abundance of *L. plantarum* was higher in additive-treated silage on D15, which indicated that additives could promote the dominance of *L. plantarum* in silage with a high DM. *Weissella* is abundant in fresh materials and tended to be dominated in the early ensiling period, while they will be replaced by more acid-tolerant *Lactobacillus* in the late ensiling period (Graf et al., 2016; Pereira et al., 2019). In our study, the high abundance of *Weissella paramesenteroides* in CK and BF treatment of silages on D7 might be due to the slow fermentation process, resulting in a lower abundance of *L. plantarum*. Furthermore, *Lactobacillus ginsenosidimutans* and *Lactobacillus spicheri* could be isolated from fermented food, and sometimes used as probiotic strains for improvement of functional foods (Gautam and Sharma, 2015; Chiş et al., 2020). However, their effects on silages should be further studied.

### Correlation Between Silage Parameters and Bacterial Community

**Figure 4:**
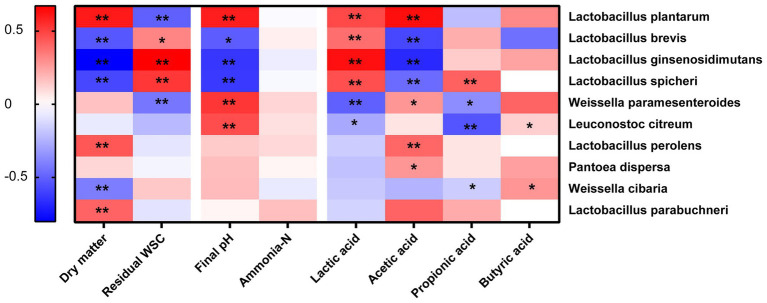
Spearman analysis between silage parameters and bacterial species. ^*^significance at *p* < 0.05; ^**^significance at *p* < 0.01.

The spearman analysis between silage parameters and bacterial species was shown in Figure 4. The results showed that residual WSC was negatively correlated to *L. plantarum* (*p* < 0.05), which confirmed that WSC was a determinant substrate for *L. plantarum* in silage fermentation (Chen et al., 2020b). *Lactobacillus* exerted a significant effect in increasing lactic acid and reducing pH in the later stage (Cai et al., 1998). Therefore, our study showed that lactic acid was positively correlated to some *Lactobacillus* genera (*L. plantarum*, *L. ginsenosidimutans*, and *L. spicheri*; *p* < 0.05). It was generally recognized that *L. plantarum* was the dominant homo-fermentative bacteria for increasing lactic acid and decreasing pH in silage fermentation. However, the final pH and acetic acid were positively correlated to *L. plantarum* (*p* < 0.05). In addition, the dry matter content was also positively correlated to *L. plantarum* in this study (*p* < 0.05). These were consistent with the results of silage quality and bacterial community of corn stalk silage after the delayed harvest, that is, the DM content and *L. plantarum* abundance increased, while the lactic acid content decreased, and the acetic acid content enhanced. The similar results were also reported by Ni et al. (2017a), who found that the lactic acid/acetic acid was lower in samples with a high DM compared with that in soybean silage samples with a low DM. That might because the high DM content was more likely cause the metabolism of hetero-fermentative *L. plantarum*, but more studies should be further conducted.

## Conclusion

Delayed harvest could restrict lactic acid fermentation, and increase acetic acid production and pH value in corn stalk silage. LP and BF additives have an ability of improving the fermentation quality of corn stalk silage through decreasing butyric acid and NH_3_-N contents. In addition, SMRT result showed that LP and BF could enhance fermentation quality of delayed harvest corn stalk by shifting bacterial community. Overall, our research confirmed that inoculated with LP or BF could be feasible ways for improving the delayed harvest corn stalk silage.

## Data Availability Statement

The datasets generated for this study can be found in Sequence Read Archive (SRA), http://www.ncbi.nlm.nih.gov/sra/, PRJNA718962.

## Author Contributions

LG, YL, PL, LC, and WG designed the study and wrote the manuscript. PL and LC performed the experiments. LG and PL conducted the statistical and bioinformatics analysis. PL and CZ were involved in the revision of the manuscript. All authors contributed to the article and approved the submitted version.

### Conflict of Interest

The authors declare that the research was conducted in the absence of any commercial or financial relationships that could be construed as a potential conflict of interest.

## Footnotes

^1^http://www.drive5.com/usearch/manual/uchime_algo.html

^2^http://drive5.com/uparse/
